# Effects of kinds of additives on fermentation quality, nutrient content, aerobic stability, and microbial community of the mixed silage of king grass and rice straw

**DOI:** 10.3389/fmicb.2024.1420022

**Published:** 2024-06-12

**Authors:** Chenchen Qiu, Kaili Yang, Xiaogao Diao, Wei Zhang, Renlong Lv, Liwen He

**Affiliations:** ^1^Sanya Institute of China Agricultural University, Sanya, Hainan, China; ^2^State Key Laboratory of Animal Nutrition and Feeding, SKLANF, College of Animal Science and Technology, China Agricultural University, Beijing, China; ^3^Tropical Crops Genetic Resources Institute, Chinese Academy of Tropical Agricultural Sciences, Haikou, Hainan, China

**Keywords:** organic acid, sucrose, king grass, rice straw, silage quality

## Abstract

To investigate the effects of kinds of additives on silage quality, the mixture of king grass and rice straw was ensiled with addition of sucrose, citric acid and malic acid at the levels of 0, 1 and 2%, being blank control (CK), citric acid groups (CA1, CA2), malic acid groups (MA1, MA2), citric acid + malic acid groups (CM1, CM2), sucrose groups (SU1, SU2), mainly focusing on fermentation quality, nutrient content, aerobic stability and microbial community of the silages. The results showed that the addition of sucrose decreased (*p* < 0.05) pH and increased the content of water soluble carbohydrate (*p* < 0.05). The sucrose groups and mixed acid groups also had a lower (*p* < 0.01) neutral detergent fiber content. The addition of citric acid and the mixed acid increased (*p* < 0.01) the aerobic stability of the silage, reduced the abundance of *Acinetobacter*, and the addition of citric acid also increased the abundance of *Lactiplantibacillus*. It is inferred that citric acid and malic acid could influence fermentation quality by inhibiting harmful bacteria and improve aerobic stability, while sucrose influenced fermentation quality by by promoting the generation of lactic acid. It is suggested that the application of citric acid, malic acid and sucrose would achieve an improvement effect on fermentation quality of the mixed silage.

## 1 Introduction

King grass (*Pennisetum purpureum* Schumacher × *P. glaucum*, *Pennisetum* genus, *Gramineae* family), a high-quality grass herbage characterized by its tall plant type, strong tiller ability and abundant leaf content with optimal growth temperature of 25–35°C, is extensively cultivated in tropical and subtropical regions (Zhao et al., 2019b). It serves as a crucial roughage source for ruminants in southern China. Due to imbalance supply in the seasons, storing king grass during summer and autumn to meet feed supply gap in winter is still necessary. Silage is a common preservation technology employed to facilitate the fermentation of green fodder in an oxygen-deprived environment, thereby maintaining its quality at a relatively consistent level (Borreani et al., 2018). The fundamental principle underlying this approach is that, in the absence of oxygen, lactic acid bacteria metabolize soluble sugars into lactic acid, which promptly reduces the pH level and inhibits the microbial spoilage of feed material (Yin et al., 2021). The high moisture content of king grass necessitates the addition of other raw materials with high water absorption capabilities to achieve desired silage quality (Zi et al., 2021), where rice straw (*Oryza sativa L*.) is one of the most effective water absorber in practical application (Kamphayae et al., 2017). Numerous studies have demonstrated favorable silage quality when rice straw is incorporated into the silage (He et al., 2020; Mu et al., 2020; Wang et al., 2022).

It is a common way to employ kinds of additives to enhance the fermentation quality when the raw materials fail to meet the conditions for ensiling fermentation. The additives commonly employed in silage can be categorized into two distinct types: fermentation promoters and fermentation inhibitors (Oladosu et al., 2016). A fermentation promoter is an additive that enhances the fermentation process of lactic acid bacteria, such as lactic acid bacteria, enzyme preparations, and saccharides (Muck et al., 2018). Fermentation inhibitors are a category of additives that serve to enhance acidity, lower the pH of silage, and impede the proliferation of undesirable microorganisms, commonly including inorganic substances (aldehydes, salts and acid–base substances) and organic acids (formic acid, acetic acid, citric acid, malic acid) (Queiroz et al., 2018). Research showed that the addition of molasses improved the fermentation quality of silage and reduced the breakdown of protein (Sutaryono et al., 2023). Sorbic acid addition significantly improved fermentation quality and aerobic stability of rice straw silage, slowing down the reduction of lactic and acetic acids content as well as the growth of yeasts and aerobic bacteria under aerobic exposure (Zhao et al., 2021). The effect of these additives may vary depending on the characteristics of silage raw material. At present, the study of kinds of additives on king grass has not been reported, and it is not yet clear which additive has a superior effect.

King grass has a high summer yield and a low winter yield due to its growth characteristics. It is essential to ensile king grass in summer for winter ruminant feed supply, but the initial quality of fresh king grass silage is poor. It is crucial to control the moisture content of king grass silage and select appropriate additives to enhance its fermentation quality. Therefore, this study aimed to examine the effects of additives (sucrose, citric acid and malic acid) on the fermentation quality, nutrient content, aerobic stability and microbial community of the mixed silage of king grass and rice straw, investigating the effects of different kinds and dosages of silage additives, and offering a reference for the preservation and quality enhancement of king grass silage.

## 2 Materials and methods

### 2.1 Silage preparation

Fresh king grass was harvested at the experimental base affiliated to the Institute of Tropical Crop Variety Resources, Chinese Academy of Tropical Agricultural Sciences, Danzhou City, Hainan Province. Then the chopped fresh king grass (1–2 cm) and air-dried rice straw (about 2 cm) were mixed in a ratio of 9:1 to adjust the moisture content of the raw materials. The mixture was ensiled with addition of citric acid (CA1, CA2), malic acid (MA1, MA2), the mixture (1,1) of citric and malic acids (CM1, CM2), sucrose (SU1, SU2) at the level of 1 and 2% on a fresh matter basis, along with blank control (CK). After thorough mixing, the materials were ensiled in lab-level polyethylene silage bags (500 g per bag), and vacuum-sealed by a house-use vacuum machine. A total of 27 bags (9 treatments×3 replicates) were individually prepared and stored at ordinary temperature (26–30°C). All silage bags were unsealed for sample Research Topic on day 30, to determine the fermentation quality, chemical composition, aerobic stability and microbial community. Additionally, citric acid and sucrose were obtained from China National Pharmaceutical Group Co. Ltd. (analytically pure). Malic acid was obtained from Hefei BASF Biotechnology Co., Ltd., with a purity of ≥99.0%. The given additive was dissolved in 30 mL distilled water and sprayed with a mini-sprayer.

### 2.2 Chemical composition and silage fermentation parameter analyses

The silage samples were oven-dried at 65°C for 72 h to determine the dry matter content (DM), and ground (1 mm sieve) for chemical composition analysis. Crude protein (CP) was measured by Kjeldahl method following the procedure of the Association of Official Analytical Chemists (AOAC, 1990). Neutral detergent fiber (NDF), acid detergent fiber (ADF) were determined by Van Soest et al. (1991). Water soluble carbohydrate (WSC) was determined using the method of Murphy (1958). The filtrate was used to measure pH with a portable pH meter, and its ammonia-N content was determined according to the method of Broderick and Kang (1980). The organic acids (lactic acid, acetic acid, propionic acid, and butyric acid) were analyzed using high-performance liquid chromatography (Chen et al., 2022).

Moreover, microbial populations were determined via the plate counting method (Madigan, 2012). In detail, 10 g of sample was added to 90 mL of sterilized saline and shaken for 1 h at 4°C to get bacterial solution, and finally the diluted stock solution was spread evenly in the medium. Lactic acid bacteria were counted after incubation on the plate of MRS agar medium at 37°C for 48 h, and yeast and mold were counted after incubation on the plate of Rose Bengal agar medium at 30°C for 72 h. The microbial population data were collected as colony forming units (CFU) and transformed to a logarithmic scale on a fresh matter basis.

### 2.3 Aerobic stability analysis

After 30 days of ensiling, each silage bag was unsealed and stirred, and then the inner temperature of silage was monitored at 10 min interval by a temperature logger with its probes inserting into the center of silage bags, along with three probes recording the environmental temperature. When the sample temperature is 2°C higher than the ambient temperature, it means that the silage has rotted and deteriorated, and the recorded time is the aerobic stabilization time (Xie et al., 2022). After 3 d of aerobic exposure, silage sample of each bag was taken to determine pH, lactic acid (LA), acetic acid (AA), propionic acid (PA) and butyric acid (BA).

### 2.4 Microbial community analysis

The silage samples collected on day 30 from the treatment groups at the 1% level and the blank group (CK) were subjected to bacterial community analysis using 16S rDNA sequencing technology. Briefly, DNA was extracted with the commercial DNA Kit (Tiangen Biotech (Beijing) Co., Ltd.) and PCR amplification was conducted with the universal primer (338F: 5’-ACTCCTACGGGAGGCAGCA-3’, 806R: 5’-GGACTACHVGGGTWTCTAAT-3’) targeting the V3-V4 region of 16S rRNA. After purification and quantification of the total of PCR amplicons, Illumina novaseq 6,000 (Illumina, Santiago CA, United States) was used for sequencing. According to quality of single nucleotide, raw data was primarily filtered by Trimmomatic (version 0.33). Primer sequences were identified using Cutadapt (version 1.9.1), PE reads were assembled using USEARCH (version 10), chimeras were removed using UCHIME (version 8.1), and high-quality reads were obtained for analysis. Sequences with similarity≥97% were clustered into the same operational taxonomic unit (OTU) by USEARCH (version 10.0), and the OTUs with reabundance <0.005% were filtered. Taxonomy annotation of the OTUs was performed based on the Naive Bayes classifier in QIIME2 using the SILVA database (release 132) with a confidence threshold of 70%. The Alpha diversity and richness (Shannon, Simpson, Chao1, Ace) were calculated and displayed by the QIIME (Version 2.15.3) and R software (R Foundation for Statistical Computing, Vienna, Austria), respectively. Beta diversity (Principal coordinate analysis [PCoA]) was determined to evaluate the degree of similarity of microbial communities from different samples using QIIME (Version 2.15.3). Furthermore, Linear Discriminant Analysis (LDA) effect size (LEfSe) was employed to test the significant taxonomic difference among groups. A logarithmic LDA score of 4.0 was set as the threshold for discriminative features. Python software (Version 2.7.8) was used to calculate the correlation coefficient through Spearman algorithm, and the correlation *p* value was calculated. The correlation between bacterial genera and fermentation parameters was visualized intuitively in the form of heatmap. The sequencing data reported in this study was archived in the Sequence Read Archive (SRA) with the accession number PRJNA1086022.

### 2.5 Statistical analysis

The collected data were analyzed using the general linear model procedure in IBM SPSS Statistics for Windows, version 26.0 (IBM, Armonk, NY, United States). Tukey’s test was used for multiple comparisons, and significant differences were determined when *p* < 0.05. The bioinformatics analysis of this study was performed with the BMK Cloud.^1^

## 3 Results

### 3.1 Chemical composition of the raw materials for silage production

Fresh king grass had a low DM content as 17.60%, a medium crude protein concentration of 8.33% DM and a high fiber content (NDF 70.69% DM, ADF 35.99% DM). As a water-absorbing agent for king grass, rice straw had a higher DM content (88.26%) and a lower CP content (3.43% DM), along with higher contents of NDF (72.30% DM) and ADF (39.33% DM).

### 3.2 Fermentation quality of the mixed silage of king grass and rice straw ensiled with kinds of additives

The pH, LA, AA, NH_3_-N content, LAB and yeast population of the mixed silage of king grass and rice straw are illustrated in Table 1. Compared to those of the CK, the pH values in MA2, CM1, CM2, SU1, SU2 as well as the LA content in CA2, MA2 and CM2 were significantly lower (*p* < 0.01), and the AA content of CA1 was significantly higher (*p* < 0.01). The NH_3_-N content of MA2 was significantly lower (*p* < 0.01) than that of the CK. Additionally, PA and BA were not detected in all the silages in the present study. In terms of microbial population, the population of LAB was significantly lower (*p* < 0.01) and that of yeast was significantly higher (*p* < 0.01) in SU1 and SU2 than those in the CK, with molds not detected (< 4 Lg CFU/g FM) in all the silages.

**Table 1:** Effect of kinds of additives on the fermentation quality of the mixed silage of king grass and rice straw.

Items	pH	LA (% DM)	AA (% DM)	NH_3_-N (% DM)	LAB (lg CFU/g FM)	Yeast (lg CFU/g FM)
CK	4.06ab	5.08ab	1.21bc	0.43abc	6.62a	< 4
CA1	4.23a	3.45bcd	2.65a	0.50a	6.93a	< 4
CA2	3.97bc	1.19d	2.28ab	0.37abcd	7.04a	< 4
MA1	4.01bc	3.21bcd	1.10c	0.39abcd	7.07a	< 4
MA2	3.58e	1.71 cd	1.01c	0.19d	6.44a	4.67b
CM1	3.73de	4.19abc	1.08c	0.23 cd	6.38a	4.59bc
CM2	3.70de	2.19 cd	1.25bc	0.27bcd	6.97a	4.10 cd
SU1	3.84 cd	5.73ab	0.90c	0.44ab	5.10b	5.88a
SU2	3.76de	6.38a	1.08c	0.34abcd	5.28b	5.83a
SEM	0.06	0.77	0.33	0.06	0.16	0.15
*p-*value	< 0.01	< 0.01	< 0.01	< 0.01	< 0.01	< 0.01

LA, lactic acid; AA, acetic acid; propionic acid and butyric acid were not detected (The lower detection limits for propionic acid and butyric acid are 0.1786 mmol/L and 0.0585 mmol/L, respectively); NH_3_-N, ammonia nitrogen; LAB, lactic acid bacteria; SEM, standard error of the means; Means with different lowercase letters in the same column differ at *p* < 0.05.

### 3.3 Nutrient composition of the mixed silage of king grass and rice straw ensiled with kinds of additives

The DM, CP, WSC, NDF and ADF content of the mixed silage of king grass and rice straw are showed in Table 2. There was no significant difference (*p* > 0.05) in DM and ADF content among all the groups. Compared with that in the CK, the CP content was significantly higher (*p* < 0.01) in CM2. The WSC content was higher (*p* < 0.01) in MA2, CM2 and SU2 than that in the CK. The NDF contents of MA2, CM2, SU1 and SU2 were significantly lower (*p* < 0.01) than that of the CK.

**Table 2:** Effect of kinds of additives on nutrient composition of the mixed silage of king grass and rice straw.

Items	DM (%FM)	CP (%DM)	WSC (%DM)	NDF (%DM)	ADF (%DM)
CK	21.60	6.41b	0.65c	66.89a	36.15
CA1	22.52	7.00ab	0.74bc	66.58a	36.94
CA2	22.92	6.94ab	1.30abc	65.86a	35.80
MA1	22.70	6.88ab	1.05abc	65.43ab	36.01
MA2	23.38	6.71b	1.37ab	62.40bc	33.31
CM1	23.76	7.04ab	0.87abc	66.21a	35.77
CM2	24.28	7.52a	1.36ab	62.36bc	33.34
SU1	24.35	7.13ab	1.01abc	62.23bc	33.74
SU2	24.15	6.73b	1.54a	61.83c	33.86
SEM	1.52	0.21	0.20	0.92	1.42
*p*-value	0.64	< 0.01	< 0.01	< 0.01	0.11

DM, dry matter; FW, fresh matter; CP, crude protein; WSC, water soluble carbohydrate; NDF, neutral detergent fiber; ADF, SEM, standard error of the means; Means with different lowercase letters in the same column differ at *p* < 0.05.

### 3.4 Aerobic stability of the mixed silage of king grass and rice straw ensiled with kinds of additives

The aerobic stability and fermentation parameters in 3 days aerobic exposure of the mixed king grass and rice straw silage are demonstrated in Table 3. The aerobic stability of CA1, CA2, MA2, CM1 and CM2 was significantly higher (*p* < 0.01) than that of the CK, but that of SU1 was significantly lower (*p* < 0.05). After 3 days aerobic exposure, the pH values of all silage groups rose exceed 0.5, and that of all additive-added groups was numerically lower than that of the CK, where the pH value of CM2 was lower than that of MA1. LA content of all additive-added groups except for MA1 and MA2 was numerically higher than that of the CK, and LA content of SU1 was significantly higher (*P*<0.01) than those of MA1 and MA2. The AA content of CA2 was significantly higher (*p* < 0.01) than that in the CK.

**Table 3:** Aerobic stability of the mixed silage of king grass and rice straw and its fermentation parameters in 3 days aerobic exposure.

Items	Aerobic stability (h)	pH	LA (%DM)	AA (%DM)
CK	20.90d	5.12a	0.97abc	0.04b
CA1	45.53a	4.53ab	2.60abc	1.38ab
CA2	45.36a	4.43ab	1.00abc	1.84a
MA1	23.74bcd	5.02a	0.32c	0.33ab
MA2	24.41bc	4.51ab	0.46bc	0.00b
CM1	25.24b	4.37ab	2.48abc	0.36ab
CM2	25.51b	4.22b	1.44abc	0.97ab
SU1	17.07e	4.74ab	3.34a	0.57ab
SU2	21.90 cd	4.41ab	2.82ab	0.34ab
SEM	0.84	0.22	0.95	0.43
*p*-value	< 0.01	0.02	0.04	< 0.01

LA, lactic acid; AA, acetic acid; propionic acid and butyric acid were not detected (The lower detection limits for propionic acid and butyric acid are 0.1786 mmol/L and 0.0585 mmol/L, respectively); SEM, standard error of the means; Means with different lowercase letters in the same column differ at *p* < 0.05.

### 3.5 Microbial community of the mixed silage of king grass and rice straw ensiled with kinds of additives

**Figure 1:**
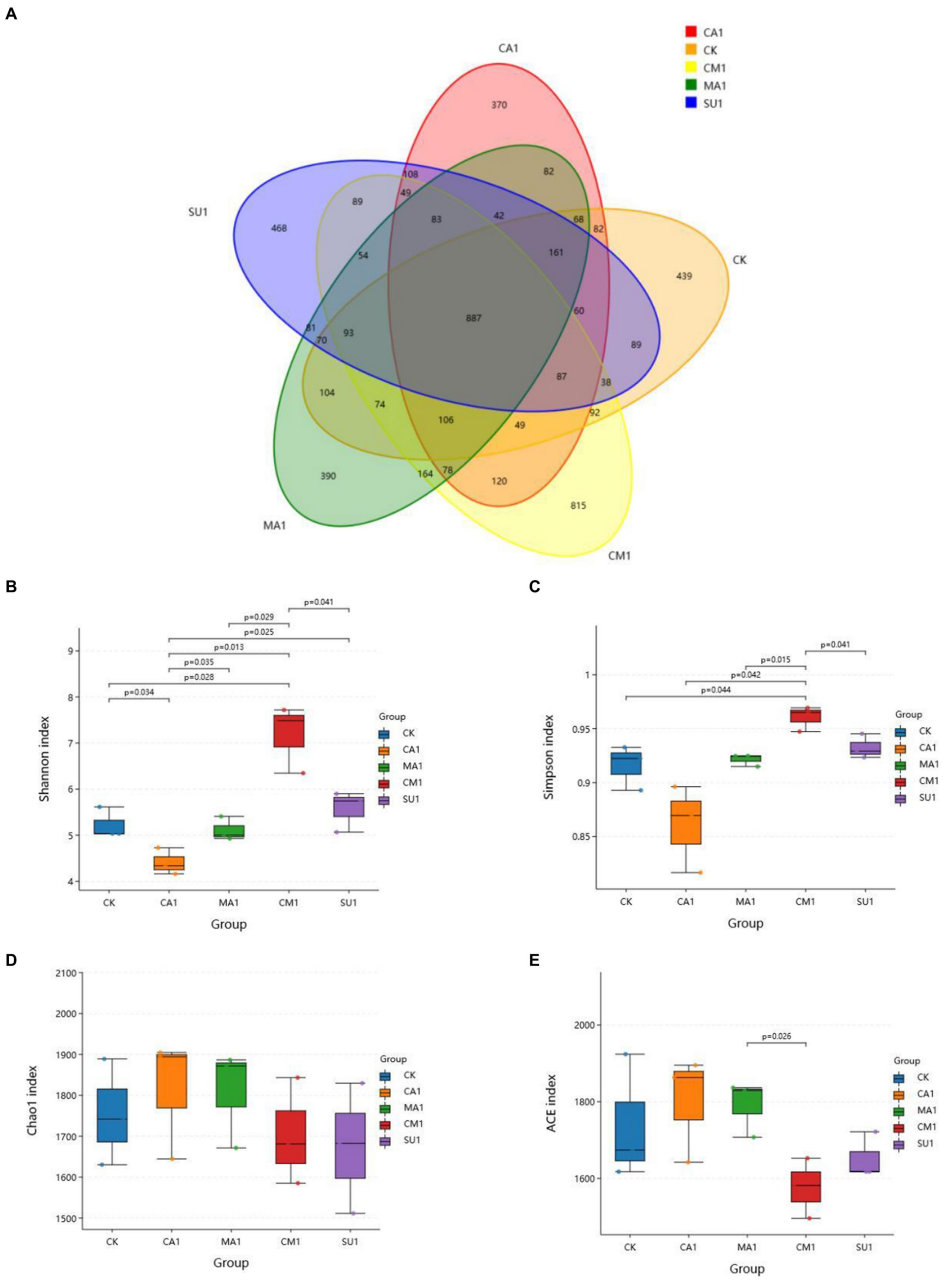
Effect of kinds of additives on the Alpha diversity of microbial community in the mixed silage of king grass and rice straw. **(A)** OTU number; **(B)** Shannon index; **(C)** Simpson index; **(D)** Chao1 index; **(E)** ACE index. (CK, blank control; CA1, 1% citric acid; MA1, 1% malic acid; CM1, 0.5% citric acid +0.5% malic acid; SU1, 1% sucrose).

**Figure 2:**
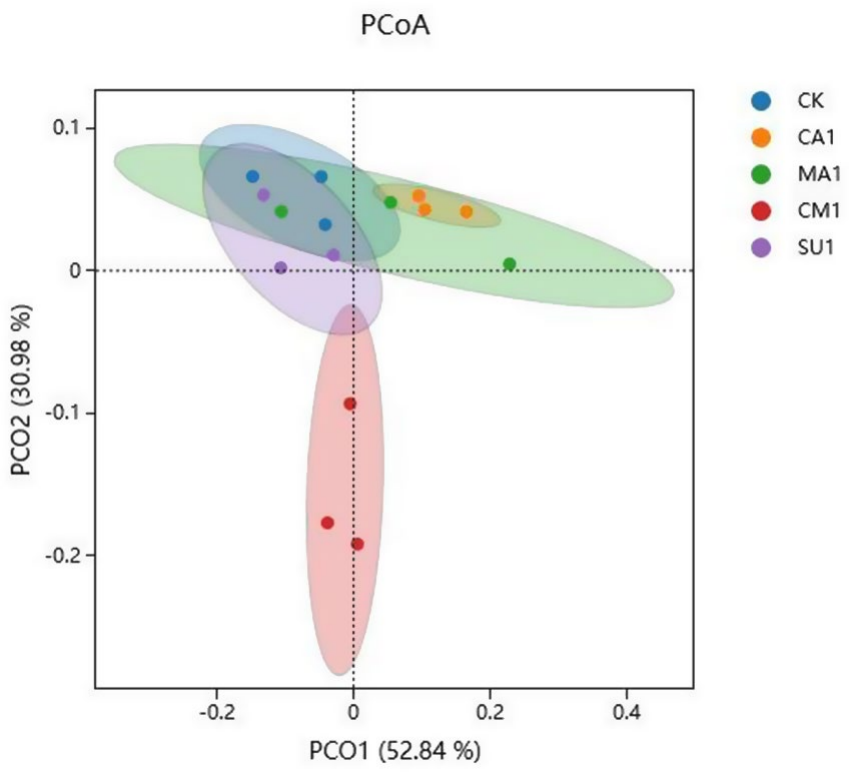
Principal coordinates analysis (PCoA, Weighted UniFrac) of bacterial community in the mixed silage of king grass and rice straw. (CK, blank control; CA1, 1% citric acid; MA1, 1% malic acid; CM1, 0.5% citric acid +0.5% malic acid; SU1, 1% sucrose).

The results of bacterial 16S rDNA sequencing of the mixed silage of king grass and rice straw ensiled with different additives showed that a total of 5,492 OTUs were identified, where 887 OTUs were shared across all treatment groups while 439, 370, 390, 815 and 468 OTUs were specific to CK, CA1, MA1, CM1, and SU1, respectively (Figure 1A). Compared to those in the CK, Shannon index and Simpson index of silage bacteria (Figures 1B,C) were increased (*p* < 0.05) in CM1 and SU1, and Chao1 index and Ace index (Figures 1D,E) were increased (*p* > 0.05) in CA1 and MA1. Principal coordinates analysis (PCoA) by weighted uniFrac method illustrated that the samples of CM1 was clearly separated from those of other groups, those of CA1 was also separated from those of the CK, but those of MA1 and SU1 were mostly overlapped with those of the CK (Figure 2).

**Figure 3:**
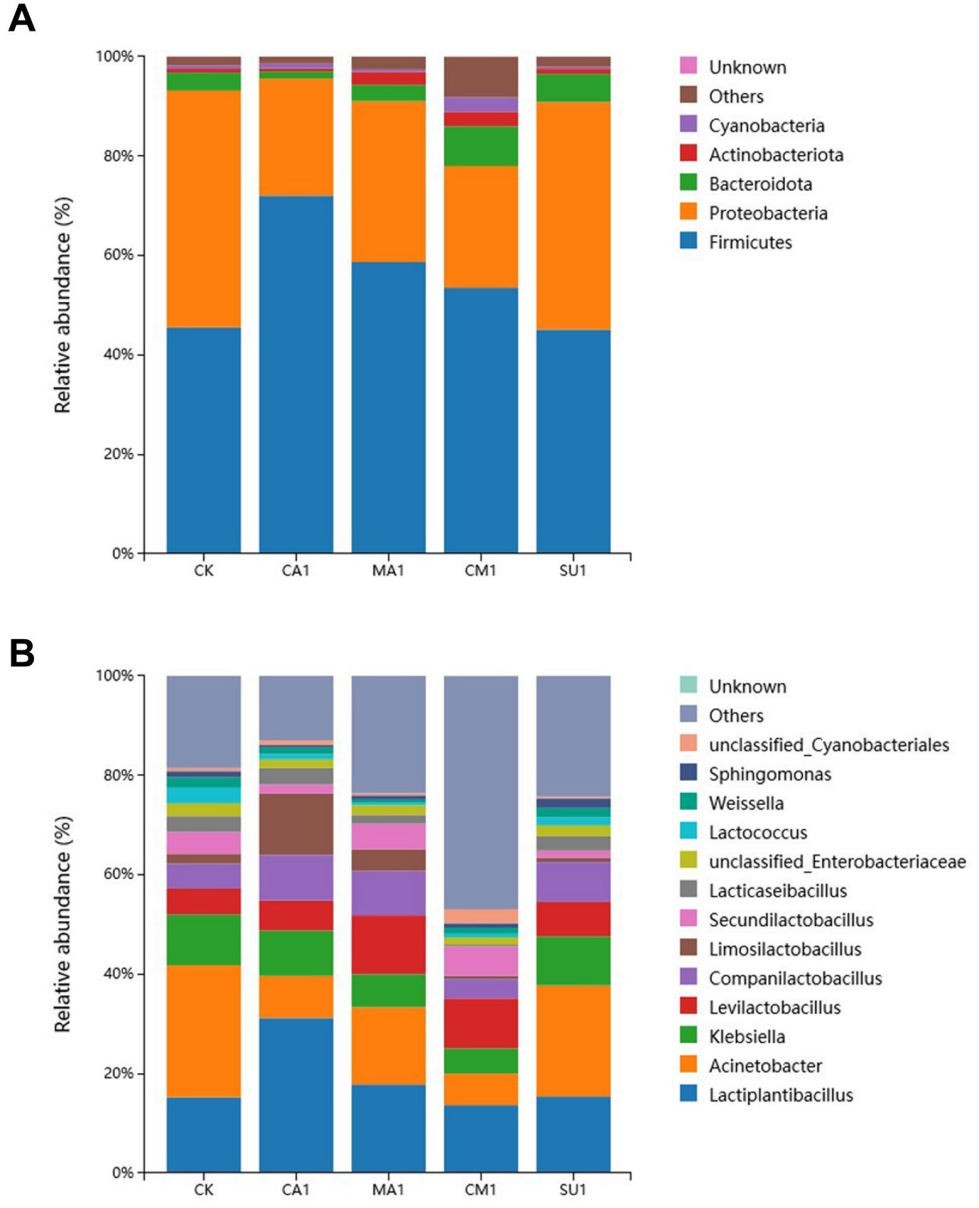
**(A)** Bacterial community at the phylum level with relative abundance greater than 1% in the mixed silage of king grass and rice straw. **(B)** Bacterial community at the genus level with relative abundance greater than 1% in the mixed silage of king grass and rice straw. (CK, blank control; CA1, 1% citric acid; MA1, 1% malic acid; CM1, 0.5% citric acid +0.5% malic acid; SU1, 1% sucrose).

The relative abundance of bacterial community in the mixed silage of king grass and rice straw is shown in Figure 3. At the phylum level (Figure 3A), Firmicutes (45.11–72.04%) and Proteobacteria (23.53–47.66%) were the dominant phyla in the silage. Compared with the CK, the abundance of Firmicutes significantly increased (*p* < 0.05) while that of Proteobacteria decreased (*p* < 0.05) in CA1, MA1 and CM1. The other bacterial phyla like Bacteroidota (1.45–7.99%), Actinobacteriota (0.60–2.92%), and Cyanobacteria (0.38–2.86%) also had a high abundance in silage. At the genus level (Figure 3B), the shared dominant genera in each treatment were *Lactiplantibacillus* (13.67–31.20%), *Acinetobacter* (6.34–26.53%), *Klebsiella* (5.07–9.88%), *Levilactobacillus* (5.21–11.80%), and *Companilactobacillus* (3.92–9.14%). In addition, the genera *Limosilactobacillus* (0.64–12.37%), *Secundilactobacillus* (1.38–5.92%), *Lacticaseseibacillus* (0.33–3.31%), unclassified_*Enterobacteriaceae* (1.48–2.55%), *Lactococcus* (0.54–3.17%), *Weissella* (0.56–2.04%), *Sphingomonas* (0.45–1.89%), and unclassified_*Cyanobacteriales* (0.37–2.81%) also had a considerable abundance in silage. Compared with the CK, MA1 and CM1 were lower (*p* < 0.05) in the abundance of *Klebsiella* and *Acinetobacter* and higher (*p* < 0.05) in the abundance of *Levilactobacillus*. It is worth noting that the abundance of *Lactiplantibacillus* and *Limosilactobacillus* in CA1 was significantly higher (*p* < 0.01) than those in other groups along with a relatively lower abundance of *Acinetobacter*.

**Figure 4:**
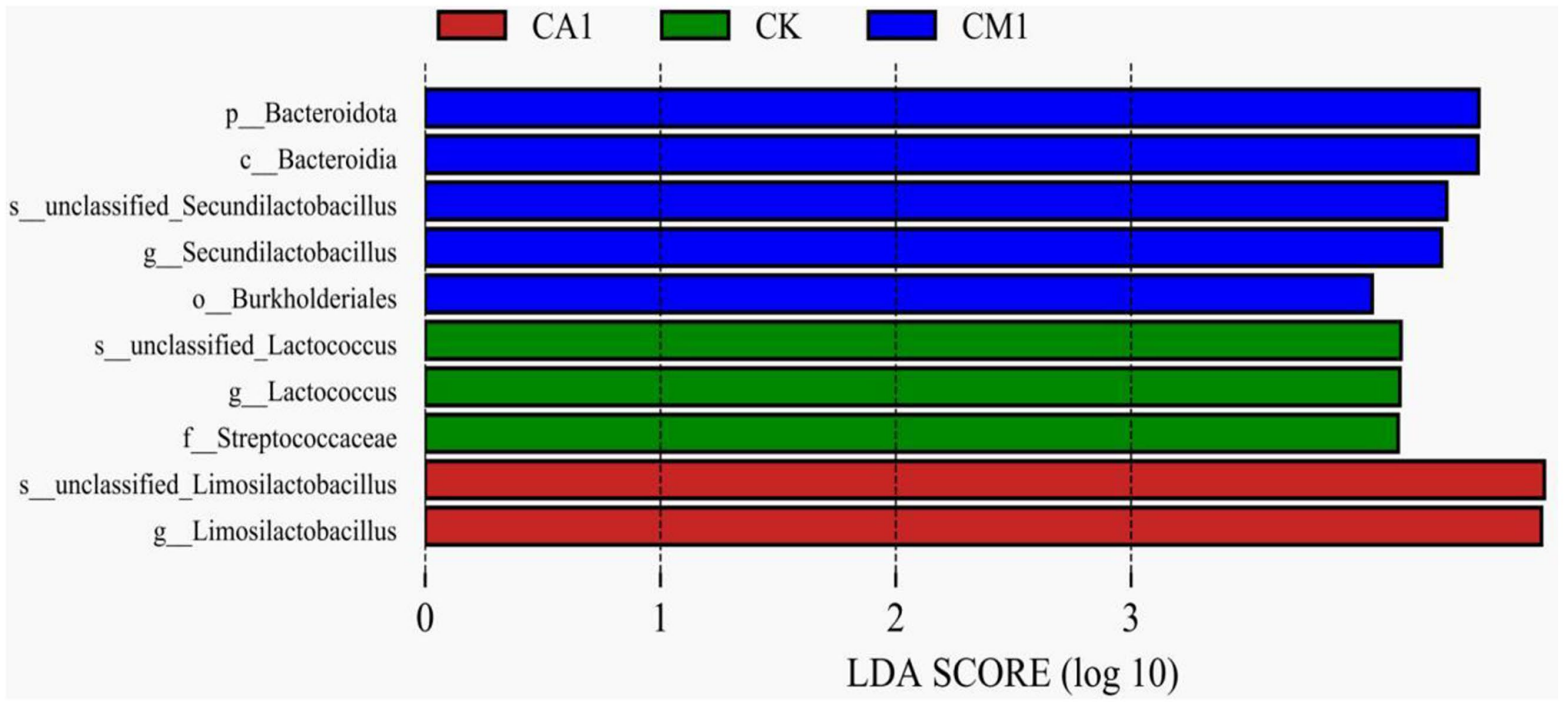
Comparison of microbial variations using the LEfSe online tool for the mixed silage of king grass and rice straw ensiled with kinds of additives. Indicator bacteria with an linear discriminant analysis (LDA) score of 4 or more in the silage bacterial community under different treatments. (CK, blank control; CA1, 1% citric acid; CM1, 0.5% citric acid +0.5% malic acid).

The LEfSe analysis (Figure 4) was utilized to explore specific bacterial species in each group [linear discriminant analysis (LDA) score > 4.0]. In detail, CK was enriched with Streptococcaceae and Unclassified_*Lactococcus*, CA1 was enriched with unclassified_*Limosilactobacillus*, and CM1 was enriched with Burkholderiales, *Bacteroidota*, and unclassified_*Secundilactobacillus*. It’s worth noting that no bacteria were enriched in MA1 and SU1.

**Figure 5:**
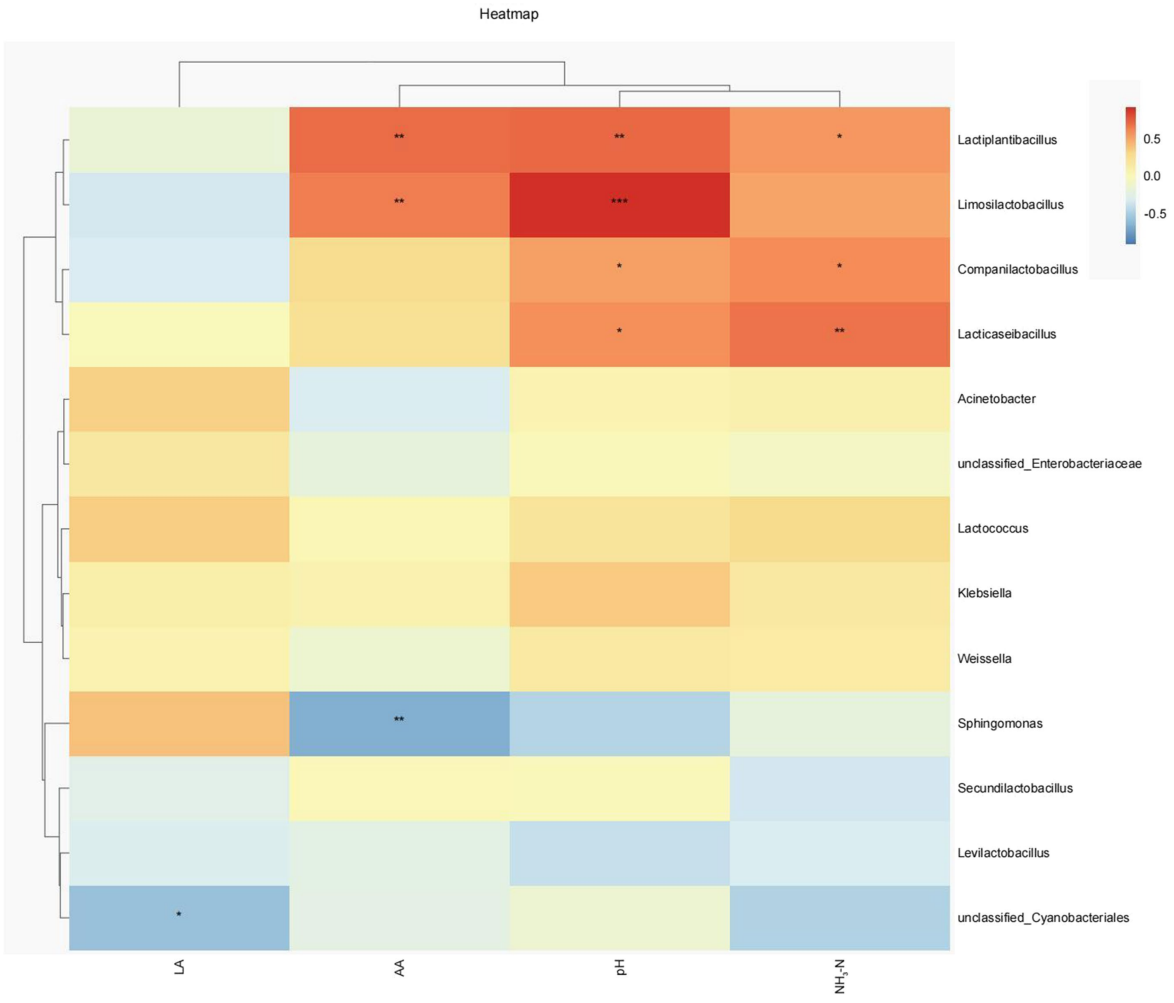
Heatmaps of Spearman’s correlations between the relative abundance of dominant genera and fermentation parameters. Red represents a positive correlation, while blue represents a negative correlation. LA, lactic acid; AA, acetic acid; NH_3_-N, ammonia nitrogen. *P < 0.05; **P < 0.01; ***P < 0.001.

Spearman correlation clustering method was used to explore the correlation between microbial communities (genus level) and fermentation characteristics (Figure 5). *Lactiplantinibacillus* showed a significant positive correlation with pH (*p* < 0.01), NH_3_-N (*p* < 0.05), and AA content (*p* < 0.01). *Limosilactobacillus* also had a significant positive correlation with pH (*p* < 0.001) and AA content (*p* < 0.01). *Lacticaseibacillus* and *Companilactobacillus* showed a significant positive correlation (*p* < 0.05) with pH and NH_3_-N content. Meanwhile, LA was negatively correlated with Unclassified_*Cyanobacteriales* (*p* < 0.01), while AA content was negatively correlated with *Sphingomonas* (*p* < 0.01).

## 4 Discussion

### 4.1 General characteristics of king grass

King grass, a member of the *Gramineae* family, is characterized by its high water content, elevated fiber levels, low soluble sugar content, and limited lactic acid bacteria. Directly ensiling fresh king grass can result in substantial juice loss and poor quality. Therefore, it is essential to regulate the moisture content of king grass and incorporate suitable additives during actual production. The primary focus for king grass should be on promoting fiber degradation and enhancing utilization efficiency. This can be achieved by either reducing the pH during silage to inhibit harmful bacterial growth or increasing the soluble carbohydrate content in king grass to provide an improved fermentation substrate for lactic acid bacteria. Consequently, selecting appropriate additives plays a critical role in enhancing the quality of king grass silage.

### 4.2 Fermentation quality of the mixed silage of king grass and rice straw ensiled with kinds of additives

pH is an important parameter reflecting the quality of silage fermentation and is usually related to the LA content in silage (Kung et al., 2018). In the present study, the pH level of the sucrose groups were found to be significantly lower than that of the CK, and the LA content in sucrose groups was higher than that of the CK. This was because the addition of sucrose increases the content of fermentation substrates in silage, leading to an increase in the lactic acid content produced by microbial fermentation. The increase in lactic acid content reduces the pH of silage. Consistently, Kang et al. (2021) demonstrated that the incorporation of sucrose into alfalfa silage resulted in an elevation of lactic acid levels and a decrease in silage pH. However, the pH value and LA content of the organic acid groups decreased with the increase of organic acid concentration, which was consistent with the research results of Ke et al. (2017). This is because high concentrations of citric acid and malic acid may inhibit the growth and fermentation of lactic acid bacteria, thereby reducing the content of LA. According to Borreani et al. (2018), lactic acid bacteria can decompose 2 mol citric acid into 1 mol LA, 3 mol AA, and 3 mol CO_2_. It might give the reason why difference in AA content was found between the citric acid groups (CA1 and CA2) and the CK, being the breakdown of citric acid into AA by lactic acid bacteria.

NH_3_-N content is an important index to evaluate the fermentation quality of silage. NH_3_-N is usually produced by the decomposition of protein in silage by plant protease and microbial fermentation, and this index is negatively correlated with the quality of silage (Blajman et al., 2018). The result of this study revealed that NH_3_-N concentration in the citric acid and malic acid groups was lower relative to that in the CK, in line with Ke et al. (2017) showing that the addition of citric acid and malic acid in alfalfa silage resulted in a decrease of NH_3_-N content. It is probably because the low pH environment provided by organic acids along with their antibacterial activity can inhibit the activity of plant protease and microbial activity, thereby reducing the production of NH_3_-N.

In general, yeast is the original microorganism that initiates aerobic deterioration, and when pH rises to a certain level, molds and other microorganisms begin to multiply and further consume the nutrients in silage, resulting in a significant loss of nutrients (Blajman et al., 2018). This study showed that the number of lactic acid bacteria in SU1 was lower and that of yeast was higher than that in the CK. This was because higher WSC content in sucrose groups might be benefit for the rapid multiplication of aerobic microorganisms such as yeasts, which would inhibit the proliferation of lactic acid bacteria such as lactobacilli and accelerate aerobic spoilage of the silage. Ke et al. (2018) reported that the addition of citric acid and malic acid to alfalfa silage increased the number of yeast in the silage, which was because citric acid and malic acid can be used as carbon sources for the growth and propagation of some yeast strains (Seo et al., 2007). Inconsistently, the population of yeast in the citric acid treatment groups was in the same level with the CK in the present study. This may be related to the higher content of AA in the citric acid treatment group, which has a strong antifungal ability and can inhibit the propagation of fungi by changing the permeability of microbial cells (Wilkinson and Davies, 2013).

### 4.3 Nutrient composition of the mixed silage of king grass and rice straw ensiled with kinds of additives

Crude protein (CP) serves as a significant indicator for evaluating the nutritional quality of silage. In the present study, the CP content of CM2 was significantly higher than that of the CK, probably because the higher concentration of organic acid inhibited the degradation of proteins by undesirable microorganisms and plant protease (Da et al., 2015). whereby potential nitrogen losses when drying decreased (lower NH_3_-N content). The WSC content is an important factor in determining the success of silage fermentation, and can provide a carbon source for the proliferation of lactic acid bacteria (Conaghan et al., 2010). In this study, the WSC content in silage of all additive-added groups was higher than that of the CK, indicating that fermentation substrate became more abundant due to the application of these additives. The WSC content of the organic acid groups increased with the increase of acid concentration, which might be because citric acid and malic acid could provide additional fermentation substrates for microbial fermentation as aforementioned (Ke et al., 2022). Meanwhile, MA1, MA2, CM1, and CM2 contained lower NDF and ADF contents, which may be related to acid degradation caused by the low pH environment of plant cell walls (Pessoa et al., 1997). Sucrose is commonly used as a nutritional additive to increase WSC in silage, and the reduction of NDF and ADF contents in the sucrose groups may be due to the ability of sucrose to promote acid hydrolysis of plant cells (Ni et al., 2017), in line with the results of Zhao et al. (2019a) reporting that molasses addition reduced the NDF and ADF contents of rice straw silage.

### 4.4 Aerobic stability of the mixed silage of king grass and rice straw ensiled with kinds of additives

Aerobic stability is a critical factor influencing the final feeding quality of silage. After exposure to the air, the growth and multiplication of aerobic microorganisms would cause the loss of nutrients and spoilage of silage, resulting in abnormal increase of temperature and pH (Tennant et al., 2017). Therefore, aerobic stability refers to the ability of silage to remain fresh and sour-smelling after contact with oxygen without an increase in both pH and temperature (Yuan et al., 2017). In the present study, the aerobic stability of SU1 was significantly lower than that of the CK. This was related to the higher WSC content and the lower AA content in the sucrose groups. The high WSC content causes rapid proliferation of aerobic bacteria, leading to aerobic spoilage. A higher content of AA can inhibit the growth of undesirable microorganisms and improve the aerobic stability of silage (Wang et al., 2018). It might also be the reason why the aerobic stability of citric acid treatment groups was significantly higher than that of other groups. Compared to the corresponding fermentation parameters on the 30th day of ensiling (Table 1), after 3 d of aerobic exposure, the fluctuation of pH of all groups exceed 0.5, and the LA and AA contents decreased to lower level, which indicated that fermentation products had been remarkably altered and aerobic spoilage of silage had occurred.

### 4.5 Microbial community of mixed silage of king grass and rice straw with kinds of additives

Making clear the alteration of bacterial community in silage would help clarify the variation mechanism of silage quality and its regulation. Nowadays, 16S rDNA sequencing technology is commonly used to investigate the diversity of bacterial community. Shannon and Simpson indices are commonly employed in quantifying species diversity, while Chao1 and Ace indices are utilized to assess species richness. Higher values of Shannon index and Simpson index indicate greater species diversity within the samples (Zhao et al., 2022). In the current study, the inclusion of mixed acid (CM1) resulted in an augmentation of Shannon and Simpson indices of bacterial community, while the single application of citric acid (CA1) or malic acid (MA1) reduced Shannon index. The possible reason is that there is a synergistic effect between these two organic acids, thus having the potential to increase the diversity and abundance of bacterial species in silage.

At the phylum level, the dominant phyla in the mixed silage of king grass and rice straw were Firmicutes and Proteobacteria, which aligned with the findings in previous studies (Liu M. et al., 2022; Zhang et al., 2023). The phylum Firmicutes contains numerous lactic acid bacteria that are actively involved in the process of fermentation, which plays a crucial role in the production of lactic acid during the later stage of ensiling (Zhao et al., 2017). The bacteria belonging to Proteobacteria have been found to exert a positive influence on protein degradation and can facilitate the production of NH_3_-N, which has adverse effects on silage preservation (Ridwan et al., 2023). In the present study, the addition of sucrose had little effect on Firmicutes and Proteobacteria of the silage, while the addition of citric acid and malic acid significantly increased the abundance of Firmicutes and decreased the abundance of Proteobacteria. Fu et al. (2022) reported similar results in ryegrass silage. It is indicated that citric acid and malic acid can inhibit the proliferation of undesirable bacteria in silage.

*Acinetobacter* is an aerobic bacterium (Howard et al., 2012) and some *Acinetobacter* can survive in the anaerobic environment in the presence of acetic acid, but require carbohydrate as an energy source, resulting in DM loss (Muraro et al., 2021). *Acinetobacter* is generally considered to be a silage-unfriendly bacterium (Liu et al., 2019). In the present study, *Acinetobacter* was the dominant genus in the sucrose-supplemented groups and the CK, which might be related to their higher AA and WSC contents. Citric acid and malic acid supplementation increased the abundance of *Lactiplantibacillus* and decreased the abundance of *Acinetobacter*. *Lactiplantibacillus* was once named *Lactobacillus* (Zheng et al., 2020). The study of Lv et al. (2020) reported that the addition of citric acid to *Amomum villosum* silage can promote the abundance of *Lactobacillus*, probably because the addition of organic acid can provide energy for *Lactobacillus* and promote its proliferation. In addition, the acidic environment formed by the addition of low concentrations of organic acids also inhibited the reproduction of undesirable microorganisms and indirectly promoted the growth of *Lactobacillus* (Zi et al., 2021). *Klebsiella* is a bacteria that is detrimental to silage and can cause aerobic spoilage of silage (Qiu et al., 2024). In this study, the combination of citric acid and malic acid showed a trend toward reducing *Klebsiella* abundance. This may be related to the synergistic effect of the two acids. *Levilactobacillus* is a type of heterofermentative lactic acid bacteria that plays an important role in promoting the production of lactic acid and reducing pH in silage (Fu et al., 2022). This study found that the addition of malic acid (MA1 and CM1) significantly increased the abundance of *Levilactobacillus*, indicating that the acidic environment created by malic acid promoted the heterofermentative mode of silage.

The LEfSe method was employed to assess variations in microbial communities among silage samples subjected to different treatments. The findings revealed discernible differences in species abundance. Fu et al. (2022) proposed that distinct bacterial species give rise to diverse bacterial metabolic pathways, leading to the production of varied metabolites during the process of silage preservation. Wang et al. (2019) revealed a significant correlation between the microbial community and fermentation quality of silage. In this study, different treatment groups enriched distinct microbial communities. Bacteroidota is often associated with the degradation of proteins and carbohydrates (Ma et al., 2023), which may result in the CM1 having poorer nutritional quality. Unclassified_*Lactococcus* is commonly found in naturally fermented silage and plays an important role in the accumulation of lactic acid and the decrease of pH throughout the ensiling process (Lu et al., 2021). Similarly, Unclassified_*Lactococcus* was enriched in the CK, indicating that these bacteria might be susceptible to the treatments of silage. The abundance of *Limosilactobacillus* in CA1 was significantly higher than that in other groups, and its aerobic stability was also significantly improved. This is in line with the report by Puntillo et al. (2020) that *Limosilactobacillus* can increase the aerobic stability of silage. In short, the significantly enriched microorganisms in different additive treatments in this study may serve as potential biomarkers affecting silage fermentation products and provide an idea for future silage research.

Ensiling is a multifaceted biological process, wherein a diverse array of metabolites are generated, intricately linked with microorganisms. In the present study, the abundance of *Lactiplantibacillus*, *Limosilactobacillus*, *Companilactobacillus*, and *Lacticaseibacillus* were positively correlated with pH and NH_3_-N content. In general, the NH_3_-N content in silage is closely related to its pH value. The decrease of pH will inhibit the decomposition of protein and reduce the production of NH_3_-N. It is speculated that lactic acid bacteria are sensitive to lower pH condition, thus NH_3_-N content and lactic acid bacteria had a high correlation coefficient. This is consistent with the results of Xu et al. (2022). The bacterium *Sphingomonas* exerts a deleterious impact on silage, resulting in an increase in pH level and degradation of protein (Ni et al., 2017). In this study, a significant negative correlation was found between *Sphingomonas* and AA content. Given that AA is an important organic acid for maintaining aerobic stability, it is inferred that the presence of *Sphingomonas* is associated with aerobic deterioration (Liu Y. et al., 2022). Unclassified_*Cyanobacteriales* primarily exist during the pre-silage period or in natural environments, and are gradually supplanted by other bacteria during the post-silage period (Huang Y. et al., 2022; Huang Z. et al., 2022). Unclassified_*Cyanobacteriales* were found to have a negative correlation with LA content. This is likely due to the competition for substrate between Unclassified_*Cyanobacteriales* and lactic acid bacteria, resulting in a decrease in LA content. It is suggested that some bacteria might play a core role in determining fermentation quality of the silage.

## 5 Conclusion

This study demonstrated that various additives exerted distinct effects on the mixed silage of king grass and rice straw. The incorporation of citric acid and malic acid enhanced the aerobic stability of silage, inhibited the growth of *Acinetobacter*, and facilitated the proliferation of *Lactiplantibacillus*. The inclusion of sucrose resulted in an elevation of LA content while concurrently leading to a reduction in NDF and ADF contents. It is inferred that citric acid and malic acid could influence fermentation quality by inhibiting harmful bacteria and improved aerobic stability, while sucrose influenced fermentation quality by directly providing substrates for lactic acid bacteria fermentation. It is suggested that the application of citric acid, malic acid and sucrose would achieve an improvement effect on fermentation quality of the mixed silage.

## Data availability statement

The datasets presented in this study can be found in online repositories. The names of the repository/repositories and accession number(s) can be found in at: https://www.ncbi.nlm.nih.gov/sra/, PRJNA1086022.

## Author contributions

CQ: Conceptualization, Data curation, Investigation, Writing – original draft, Writing – review & editing. KY: Methodology, Software, Investigation, Writing – review & editing. XD: Methodology, Software, Writing – review & editing. WZ: Conceptualization, Methodology, Writing – review & editing. RL: Project administration, Supervision, Visualization, Writing – review & editing. LH: Funding acquisition, Project administration, Supervision, Visualization, Writing – review & editing.

## Footnotes

^1^ https://www.biocloud.net/
